# Supplementary material for: Spatiotemporally distinct responses to mechanical forces shape the developing seed of Arabidopsis
Source: EMBO J. 2024 Jun 3;43(13):2733–58. doi: 10.1038/s44318-024-00138-w (PMC11217287; doi:10.1038/s44318-024-00138-w)
Supplement: Supplementary file 1 — Appendix [file 44318_2024_138_MOESM1_ESM.pdf]

Appendix

Spatiotemporally distinct responses to mechanical forces shape the developing seed of *Arabidopsis*

Amélie Bauer, Olivier Ali, Camille Bied, Sophie Bœuf, Simone Bovio, Adrien Delattre, Gwyneth Ingram, John F. Golz and Benoit Landrein

Table of contents :

Appendix Figure S1 p: 2

Appendix Figure S2 p: 3

Appendix Figure S3 p: 4

Appendix Figure S4 p: 5

Appendix Supplementary Methods p: 6

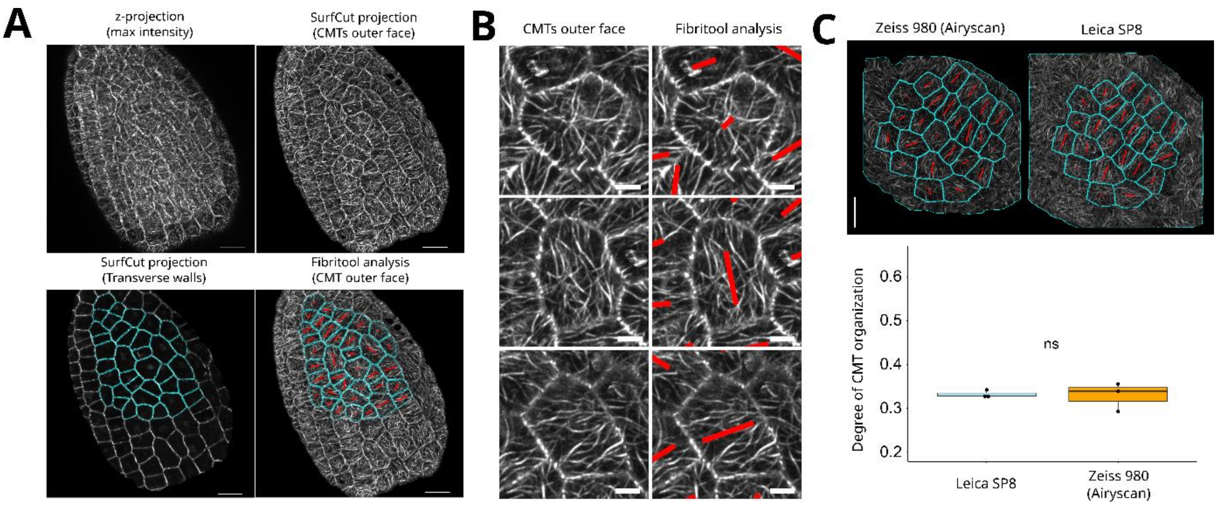

Appendix Figure S1 Analysis of CMT organization using the FibrilTool plugin

A. Process of analysing CMT organization using the CutSurf and FibrilTool plugins in ImageJ based on a confocal stack of a 2DPA seed expressing the ubiquitous CMT reporter *p35S::MAP65-1-RFP*. The SurfCut plugin generates 2D projections of the fluorescent signal at any distance from a detected surface, allowing to extract the signal of the CMTs facing transverse walls, that can be quantified or used to segment cell contours, and allowing to extract the signal of the CMTs facing the outer face (at close distance from the detected surface) or the inner face (at longer distance, provided that the reporter is expressed in only one layer) of the seed in the considered layer. The segmentation of the cell contour obtained from the projection of the signal facing transverse walls together with the projection of the signal facing the outer side of the seed can then be used by FibrilTool to measure CMT orientation and degree of organization in the outer face or the inner face of the seed in each cell. Note that this process can also be done on 2.5D surfaces obtained using the MorphographX software (as shown in panel C). Scale bars, 20 µm.

B. Close-up views on some cells from panel A showing representative CMT arrays in the outer face of the abaxial outer integument epidermis of seeds at 2DPA and the orientation (red bar) and degree of organization (length of the red bar) computed by FibrilTool.

C. Quantification of the degree of organization computed by FibrilTool based on 2.5D segmentation of the surface of the seeds with MorphographX obtained from confocal stacks of the same seed imaged with a Leica SP8 (spectral microscope) or with a Zeiss 980 Airyscan (high resolution), n= 178 cells from 3 seeds, data were compared using a bilateral Student test.

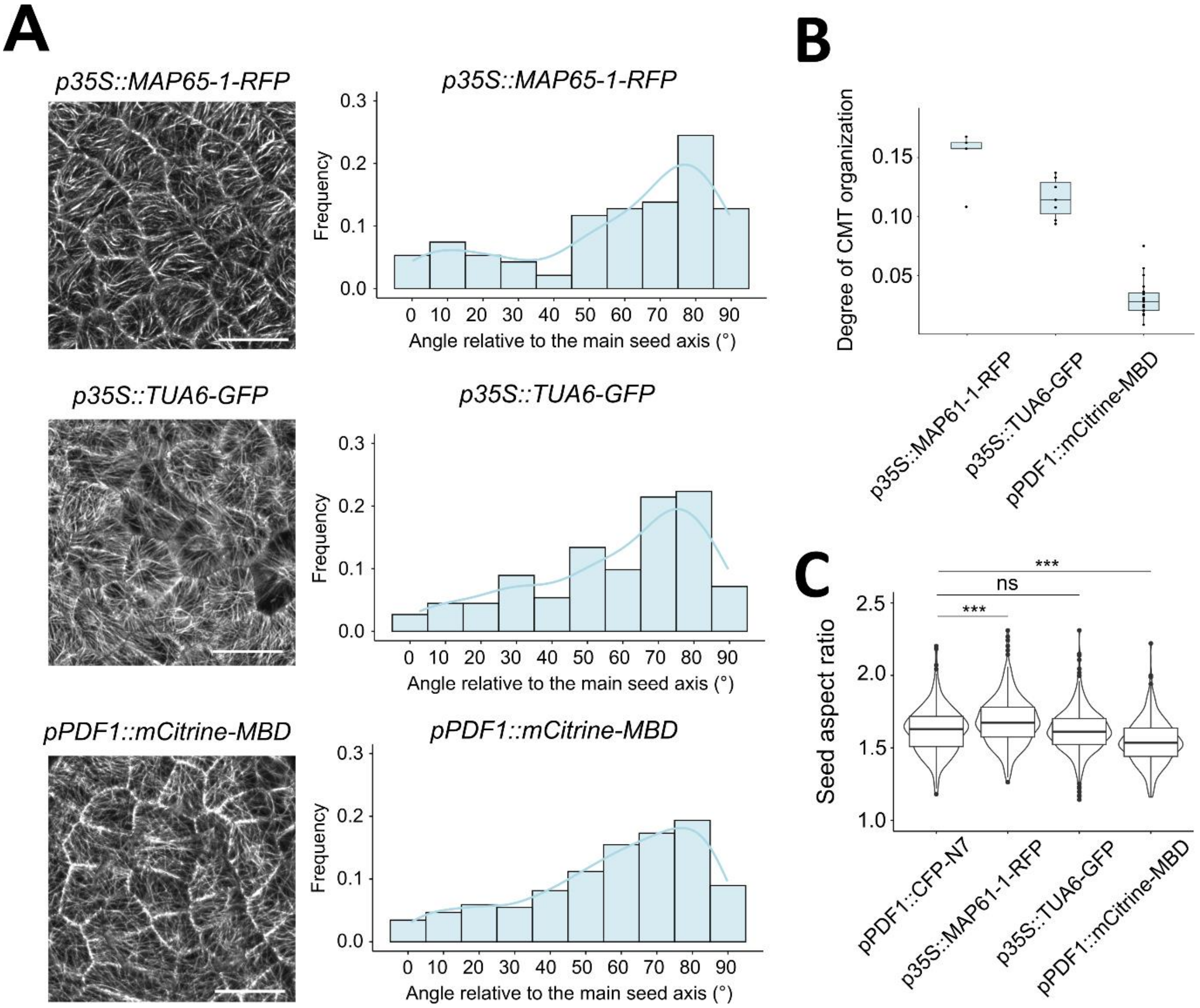

Appendix Fig. S2 Different CMT reporters show similar orientations of the CMTs in the abaxial epidermis but different degrees of organization that correlate with changes in seed shape

A. Orientation of the CMTs facing the outer side of the seed in the abaxial outer integument epidermis at 2DPA and assessed using three different CMT reporters: *p35S::MAP65-1-RFP, p35S::TUA6-GFP* and *pPDF1::mCitrine-MBD*, scale bars: 20 µm, n= 94 to 491 cells from 5 to 23 seeds, two to three independent experiments depending on the reporter.

B. Mean degree of organization of the CMTs in the outer face of the abaxial epidermis at 2DPA obtained by imaging three different CMT reporters: *p35S::MAP65-1-RFP, p35S::TUA6-GFP* and *pPDF1::mCitrine-MBD*, n= 94 to 491 cells from 5 to 23 seeds, two to three independent experiments depending on the reporter.

C. Aspect ratio of mature seeds of a control line (*pPDF1::CFP-N7*) and of the three CMT reporters presented in A and B, n=349-554 seeds, one experiment. Data were compared using Bilateral Student tests. The aspect ratio of mature seeds positively correlates with the degree of organization of the CMTs in the outer face of abaxial outer integument epidermis measured during the anisotropic growth phase.

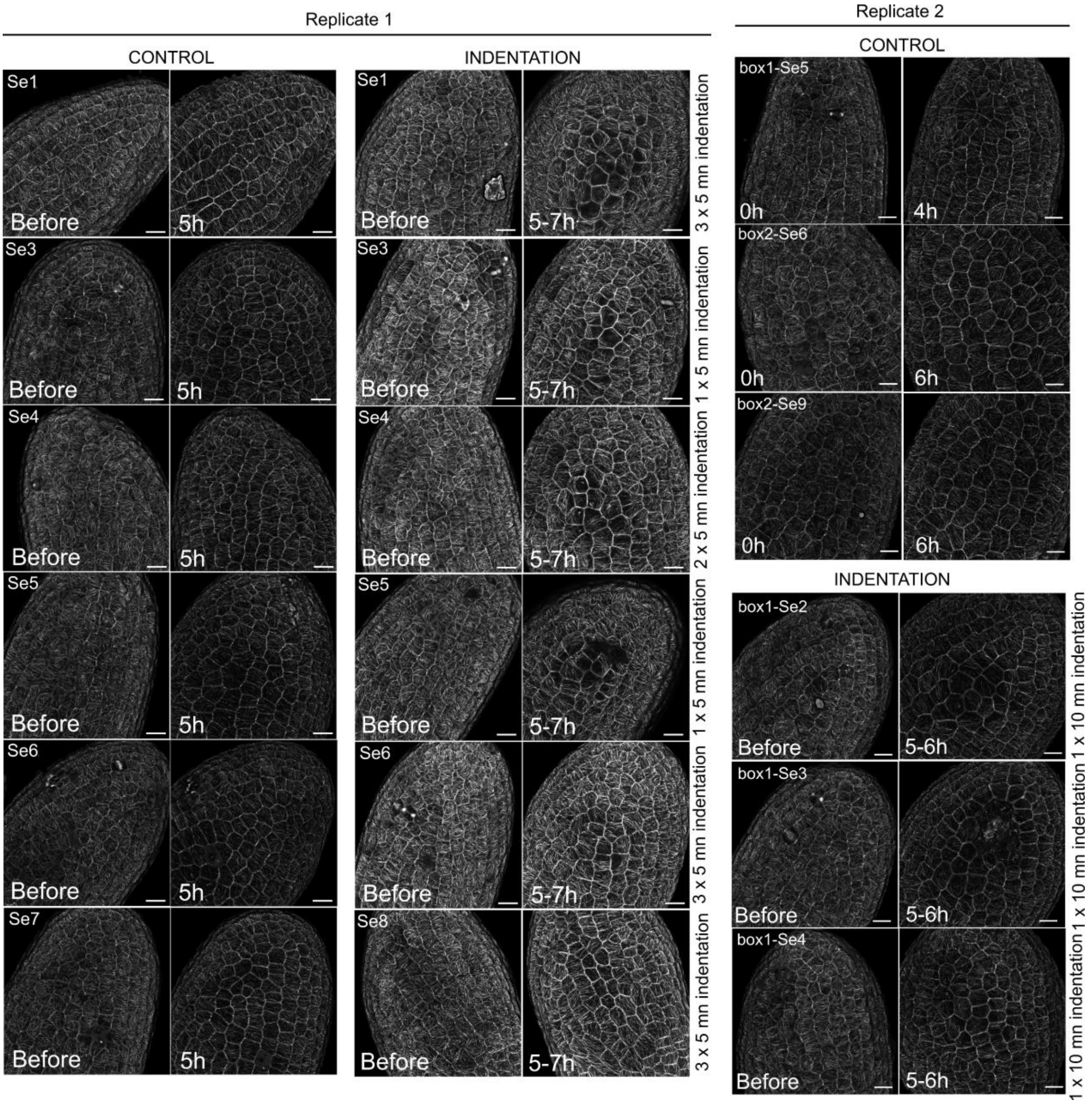

Appendix Fig. S3 Response of the CMTs to short-term indentations of 2DPA seeds

Visualization of all the replicates from the experiment showing the reorganization of the CMTs imaged using the *p35S::MAP65-1-RFP* reporter) facing the outer face of the outer integument abaxial epidermis 5 to 6h after a 20µm indentation of a 2DPA seed for one time 10 minutes or for 1 to 3 times 5 minutes (See Material and Methods for rationale). A noticeable response can be seen in 8 out of the 10 indentations that were performed.

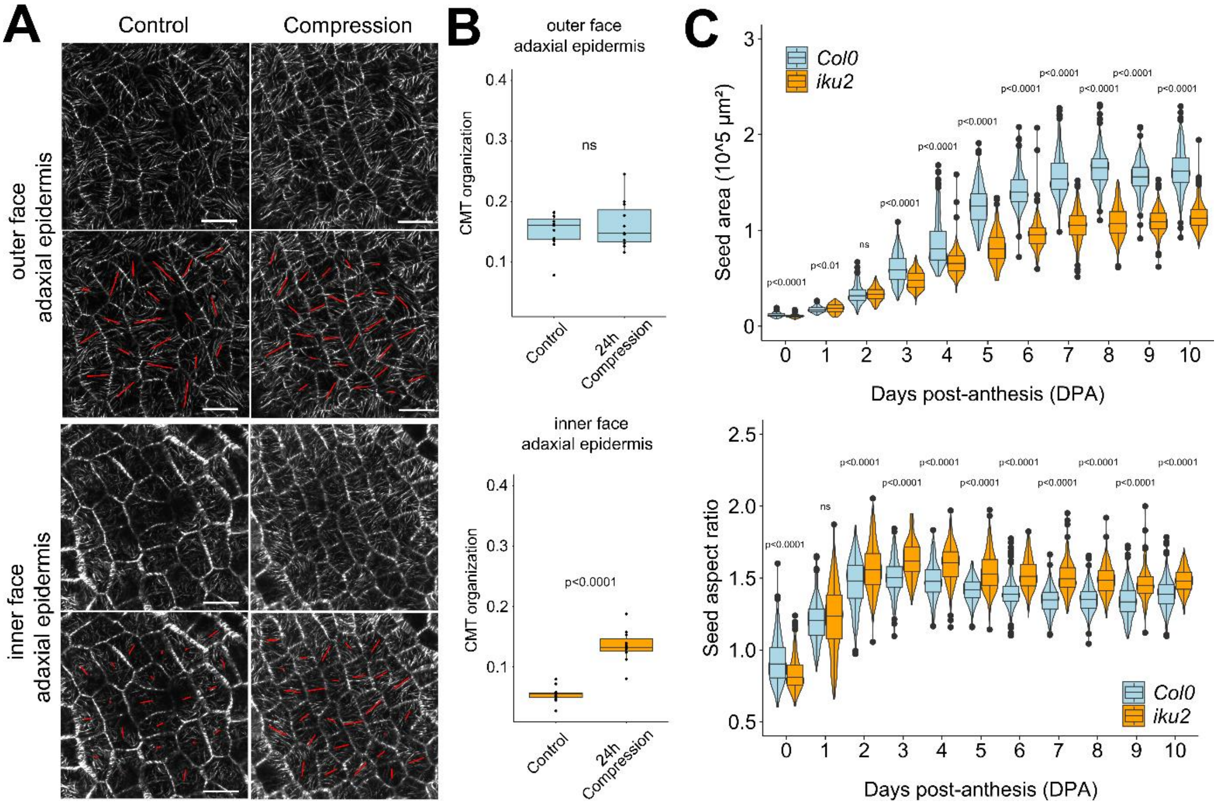

Appendix Fig. S4 CMT responses to forces in the adaxial epidermis and transition from anisotropic to isotropic growth

A-B Representative images (A) and quantification of the degree of organization (B) of the CMTs (imaged using the *pELA1::MAP65-1-mCitrine* reporter) facing the inner and the outer face of the seed in the adaxial outer integument epidermis during the isotropic growth phase (5DPA) in control seeds or in seeds whose fruits were compressed for 24h. Scale bars: 20 µm, n= 183 to 299 cells from 11 seeds per condition, two independent experiments. Data were compared using bilateral Student tests. In the boxplot representations, the midline represents the median of the data while the lower and upper limits of the box represent the first and third quartile respectively. The error bars represent the distance between the median and one and a half time the interquartile range.

C. Measurements of the area and aspect ratio of developing seeds from 0 to 10 days post-anthesis (DPA) in the WT and the endosperm-pressure defective mutant *iku2*, n= 207-313 seeds per day per genotype, three independent experiments. Data were compared using bilateral Student tests.

Appendix Supplementary Methods: Description of the simulations

Objective of the simulation campaign

The goal of the performed simulation campaign was to assess the correlation between the orientation of mechanical stresses arising onto an inflated shell and the principal directions of its curvature.

Geometrical structure

The morphomechanical context of this campaign suggested to consider shells inspired by *Arabidopsis* seeds. To that end, we performed measurements of seeds height, width and thickness and computed their mean values ({*H,W,T*}) over six seeds at two days post-anthesis (imaged using the membrane marker *LTi6b-GFP* as shown in Fig. EV1E). We approximated the *Arabidopsis* seed as an ellipsoid (S), featuring the same average aspect ratios as the considered seed population:

$$x^2 + \left(\frac{y}{w}\right)^2 + \left(\frac{z}{h}\right)^2 = 0 , \qquad (1)$$

with $w = W/T$ and $h = H/T$.

We generated a triangulated version of this ellipsoid using the BVPy python library (*1*), whose mesh generation tools are based on GMSH (*2*).

The notebook ellipsoidsgeneration.ipynb, stored with the companion gitlab reposity, was used to generate this ellipsoid. The obtained structure is recorded within the data/meshes/ subfolder of the repository.

Model description

We computed pressure-induced stress fields on the generated ellipsoid shell, using the *FiniteElement Method* (*FEM*). Since we only were interested in the surface stresses, tangent to the ellipsoid, we assumed *plane stress conditions*. The considered ellipoid was granted with a *homogeneous isotropic linear elastic* behavior, grasped by the classic Hooke's law, relating stress (σ) and strain (ε) fields:

$$\boldsymbol{\sigma} = \frac{E}{1+\nu}\left(\boldsymbol{\varepsilon} + \frac{\nu}{1-\nu}\mathrm{tr}(\boldsymbol{\varepsilon})\boldsymbol{I}_d\right) , \qquad (2)$$

where E and v respectively depicts the Young's modulus and Poisson's ratio of the ellipsoid. Assuming small deformations, we used the linear measure for strain:

$$\boldsymbol{\varepsilon} = \frac{\boldsymbol{\nabla}(\boldsymbol{v})^t + \boldsymbol{\nabla}(\boldsymbol{v})}{2} , \qquad (3)$$

where v accounts for the displacement field between the resting and current configurations.

We formalized the loading forces as a pressure-induce force field along the normal of the surface ($\hat{n}$), proportional to the pressure differential ($\Delta P > 0$) between the inner volume, encapsulated by the ellipsoid, and its surrounding:

$$f_P = \Delta P\hat{n}. \quad (4)$$

These loading forces are eventually balanced by the elastic response of the shell and the system reaches mechanical equilibrium:

$$\nabla\cdot\sigma + f_P = \mathbf{0} \text{ on } S. \quad (5)$$

This simple model features a limited number of parameters ($Y, \nu, \Delta P$). The major constrain governing the selection of their values was to keep the system in the small deformation regime, *i.e.* $||\varepsilon|| \leq 8\%$. To that end, we chose the following values:

$$Y = 1000 \text{ A.U.}, \qquad \nu = 0.5, \qquad \Delta P = 350 \text{ A.U.}$$

Implementation

Combined together eqs. (2) to (5) applied on S form a boundary value problem. We used the BVPy library (*1*), based on the FEniCS framework (*3*) to implement it in *FEM*. We used vector-valued Lagrange finite elements or degree 1 and the Petscgmres linear solver to reach convergence.

The *FEM*-based implementation is available within the stresscomputation.ipynb notebook, stored within the src/ folder of the companion gitlab repository. Results of the simulations are recorded within the data/simulationresults/ folder.

Finally, we extracted the eigenvalues and eigenvectors of the stress tensor field for each finite element of S. This has been performed within the stressanalysis.ipynb notebook, using the numpy.linalg.eigh method.

Curvature estimation

In parallel to the stress computation, we also wanted to estimate locally the curvature of the surface for each finite element. To that end, we used the Weingarten equation, defining the extrinsic curvature tensor as the derivative of the unit normal vector field:

$$\kappa = \nabla(\hat{n}). \quad (6)$$

Once this symmetric tensor field computed, we computed its eigenvectors and eigenvalues similarly to what we have done with the stress tensor field.
Finally, quantitative comparison of stress and extrinsic curvature eigenvectors and eigenvalues has been performed within the stressanalysis.ipynb notebook.

References

1. F. Gacon, C. Godin, O. Ali, *Journal of Open Source Software* 6, 2831 (2021).
2. C. Geuzaine, J. Remacle, *International Journal for Numerical Methods in Engineering* 79, 1309 (2009).
3. Logg, Mardal, Anders and, Wells, Kent-Andre and, Garth, *Automated Solution of Differential Equations by the Finite Element Method, The FEniCS Book* (2012).
