# Supplementary material for: Spatiotemporally distinct responses to mechanical forces shape the developing seed of Arabidopsis
Source: EMBO J. 2024 Jun 3;43(13):2733–58. doi: 10.1038/s44318-024-00138-w (PMC11217287; doi:10.1038/s44318-024-00138-w)
Supplement: Supplementary file 4 — Expanded View Figures [file 44318_2024_138_MOESM4_ESM.pdf]

Expanded View Figures

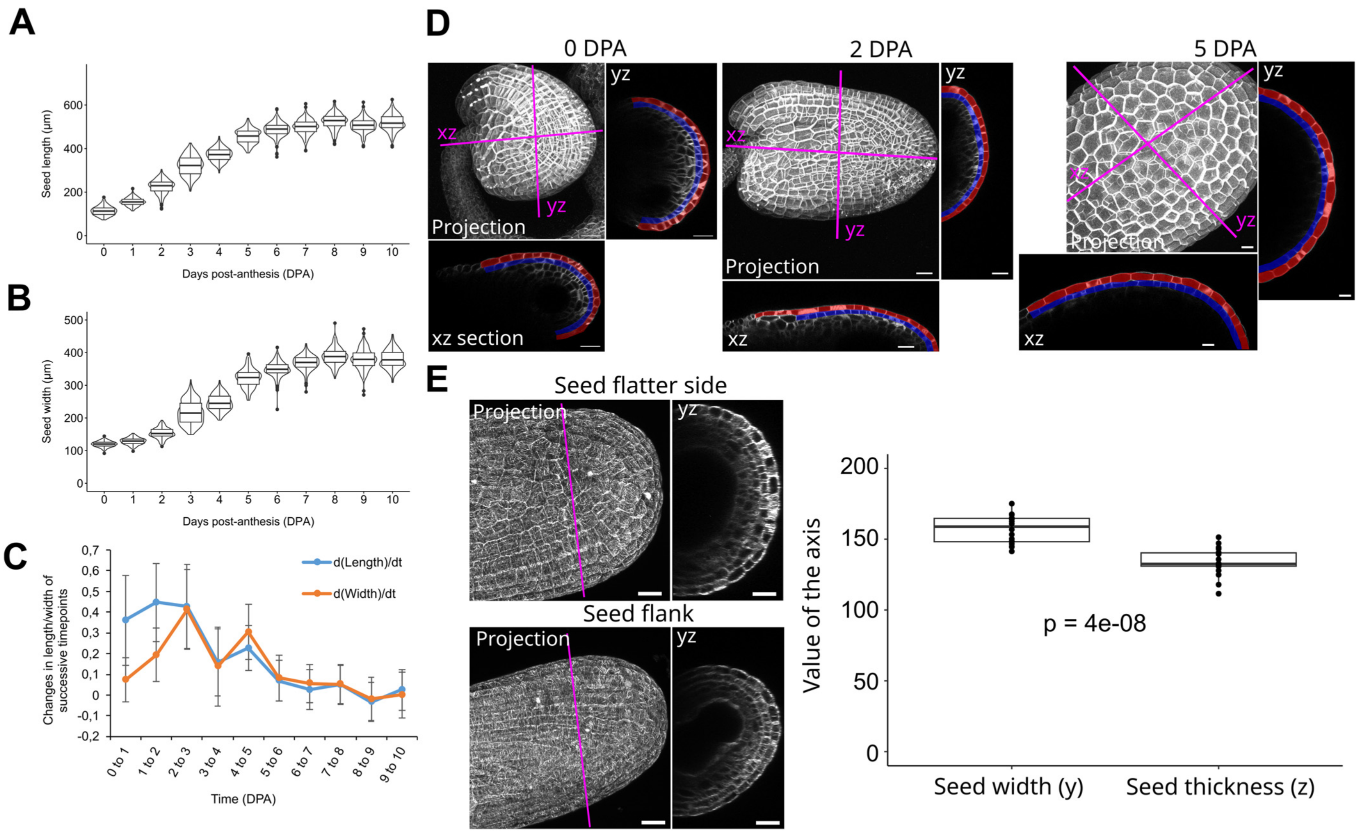

Figure EV1. Evolution of the width, length and height of developing seeds.

(**A**, **B**) Measurements of the length (**A**) and width (**B**) of WT seeds (Col-0 ecotype) from 0 to 10 days post anthesis (10DPA), $n = 180$–209 seeds per day, two independent experiments. In the boxplot representations, the midline represents the median of the data while the lower and upper limits of the box represent the first and third quartile, respectively. The error bars represent the distance between the median and one and a half time the interquartile range. (**C**) Relative changes in seed length and width over time obtained by deriving the measurements of seed length and width of (**A**, **B**). The error bars show the standard deviation of the derivative (see "Methods"). (**D**) Representative z-projection and middle sections along the width of the seed imaged using the ubiquitous membrane marker (*p35S::LTi6b-GFP)* at 0, 2 and 5DPA. The outer integument abaxial and adaxial epidermis are overlayed in red and blue, respectively. Scale bars, 20 µm. (**E**) Comparison between seed width and seed thickness obtained by manually fitting an ellipse on the seed surface of middle sections of seeds oriented on their flatter sides or on their flank, $n = 8$ to 10 seeds, two independent experiments. Scale bars, 20 µm. Data were compared using bilateral Student tests. In the boxplot representations, the midline represents the median of the data while the lower and upper limits of the box represent the first and third quartile, respectively. The error bars represent the distance between the median and one and a half time the interquartile range.

 © The Author(s)

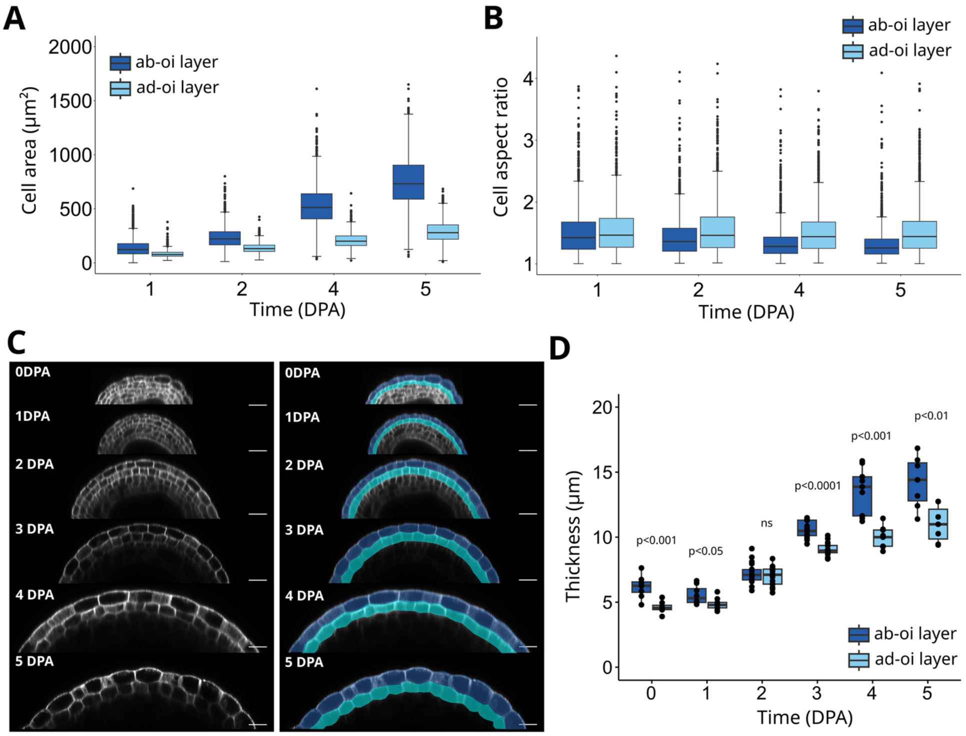

Figure EV2. Measurements of outer integument cell size and shape.

(**A**, **B**) Evolution of the size (measured as area) and shape (measured using the aspect ratio) of the cells in the abaxial and adaxial epidermis of the outer integument (DPA: Days post anthesis), 1373 to 2402 cells from 10 to 11 seeds, two independent experiments. In the boxplot representations, the midline represents the median of the data while the lower and upper limits of the box represent the first and third quartile, respectively. The error bars represent the distance between the median and one and a half time the interquartile range. (**C**) Representative middle sections along the width of the seeds (imaged using the microtubule reporter *p35S::MAP65-1-RFP)* showing the evolution of the thickness of the cells in the abaxial (overlayer in dark blue) and the adaxial (overlayed in light blue) epidermis of the outer integument. Scale bars: 20 µm. (**D**) Evolution of the thickness of the cells in the outer integument abaxial (ab-oi) and adaxial (ad-oi) epidermis from 0 to 5DPA based on manual measurements performed on sections similar to the ones presented in (**C**), $n = 45$–60 cells from 9 to 12 seeds, two independent experiments for 0, 1, 2, 3, 5,DPA, one experiment for 4DPA. Data were compared using bilateral Student tests. In the boxplot representations, the midline represents the median of the data while the lower and upper limits of the box represent the first and third quartile, respectively. The error bars represent the distance between the median and one and a half time the interquartile range.

© The Author(s)

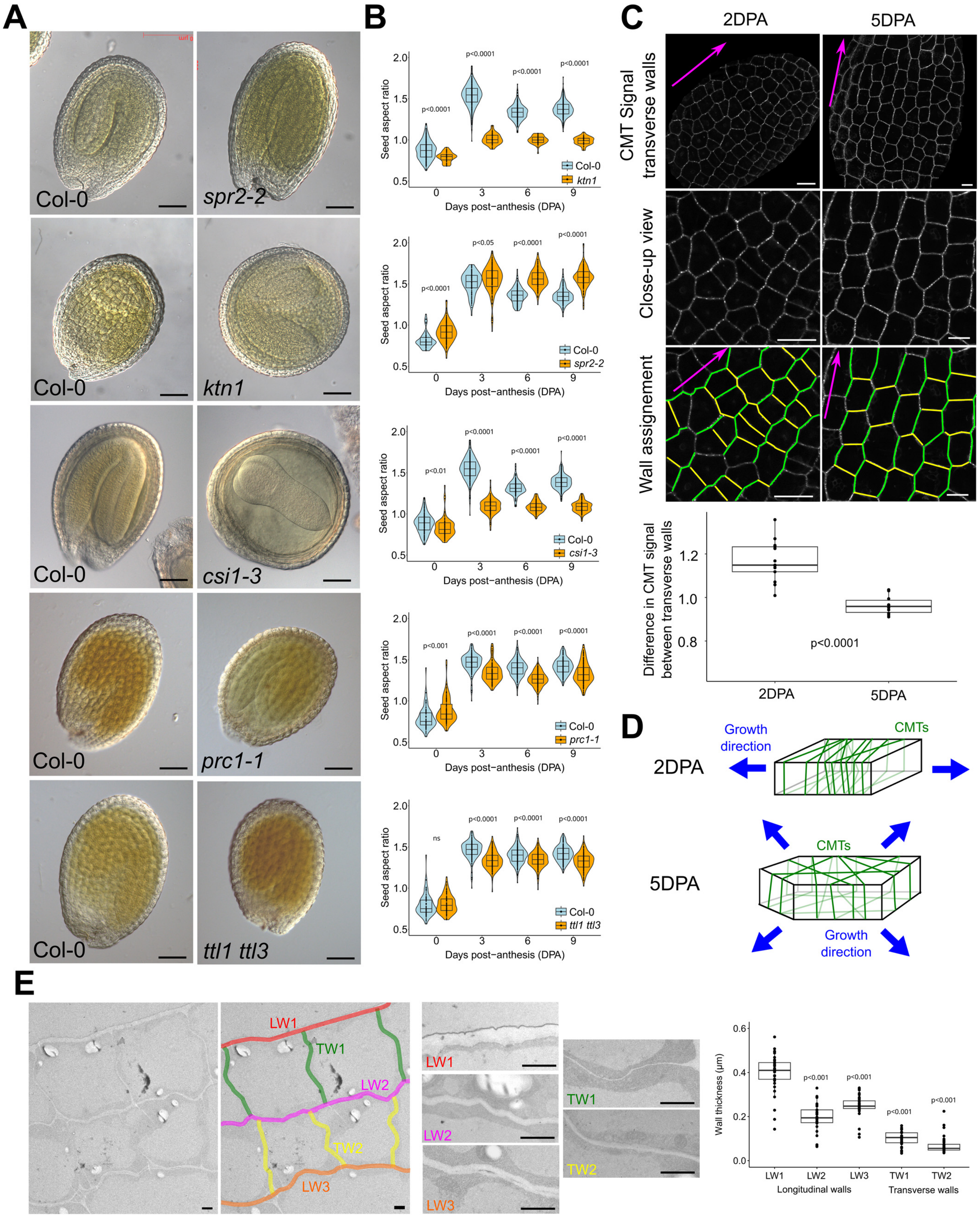
A
Col-0
spr2-2
Col-0
ktn1
Col-0
csi1-3
Col-0
prc1-1
Col-0
ttl1 ttl3
B
Seed aspect ratio
Days post−anthesis (DPA)
Col-0
ktn1
spr2-2
csi1-3
prc1-1
ttl1 ttl3
p<0.0001
p<0.05
p<0.01
p<0.001
ns
C
2DPA
5DPA
CMT Signal transverse walls
Close-up view
Wall assignement
Difference in CMT signal between transverse walls
p<0.0001
D
Growth direction
CMTs
2DPA
5DPA
E
LW1
TW1
LW2
TW2
LW3
Wall thickness (µm)
Longitudinal walls
Transverse walls
p<0.001

 © The Author(s)

Figure EV3. Mechanical control of seed elongation.

(**A**) Seed shape phenotype at 9DPA of the mutants of CMT organization and response to forces: *katanin* (*ktn1*) and *spiral2* (*spr2*), the mutants of cellulose guidance by the CMTs: *csi1-3* and *ttl1 ttl3*, and the mutant of cellulose synthase subunit: *prc1-1 (cesa6*). Scale bars: 100 µm. (**B**) Evolution of the aspect ratio of developing WT, *ktn1*, *spr2-2*, *csi1-3, ttl1 ttl3*, and *prc1-1 (cesa6)* and mutant seeds, $n = 28–366$ seeds per day per genotype, two independent experiments. Data were compared using bilateral Student tests. In the boxplot representations, the midline represents the median of the data while the lower and upper limits of the box represent the first and third quartile, respectively. The error bars represent the distance between the median and one and a half time the interquartile range. (**C**) Quantification of the CMT signal facing transverse walls in the seed coat outer integument abaxial epidermis (imaged using the *p35S::MAP65-1-RFP* reporter) as a function of the orientation of the wall relative to the main seed axis (perpendicular or parallel), $n = 100–130$ walls from 10 to 13 seeds, two independent experiments. Data were compared using a bilateral Student test. Scale bars, 10 µm. In the boxplot representations, the midline represents the median of the data while the lower and upper limits of the box represent the first and third quartile, respectively. The error bars represent the distance between the median and one and a half time the interquartile range. (**D**) Model of the organization of the CMTs in the outer integument abaxial epidermis at 2DPA and 5DPA. (**E**) TEM iImaging and quantification of the mean thickness of transverse and longitudinal walls of outer integument cells of seeds at 3DPA, $n = 32–42$ walls from 5 seeds, one experiment. All walls were compared to wall 1 using bilateral Student tests. Scale bars, 2 µm. In the boxplot representations, the midline represents the median of the data while the lower and upper limits of the box represent the first and third quartile, respectively. The error bars represent the distance between the median and one and a half time the interquartile range.

© The Author(s)

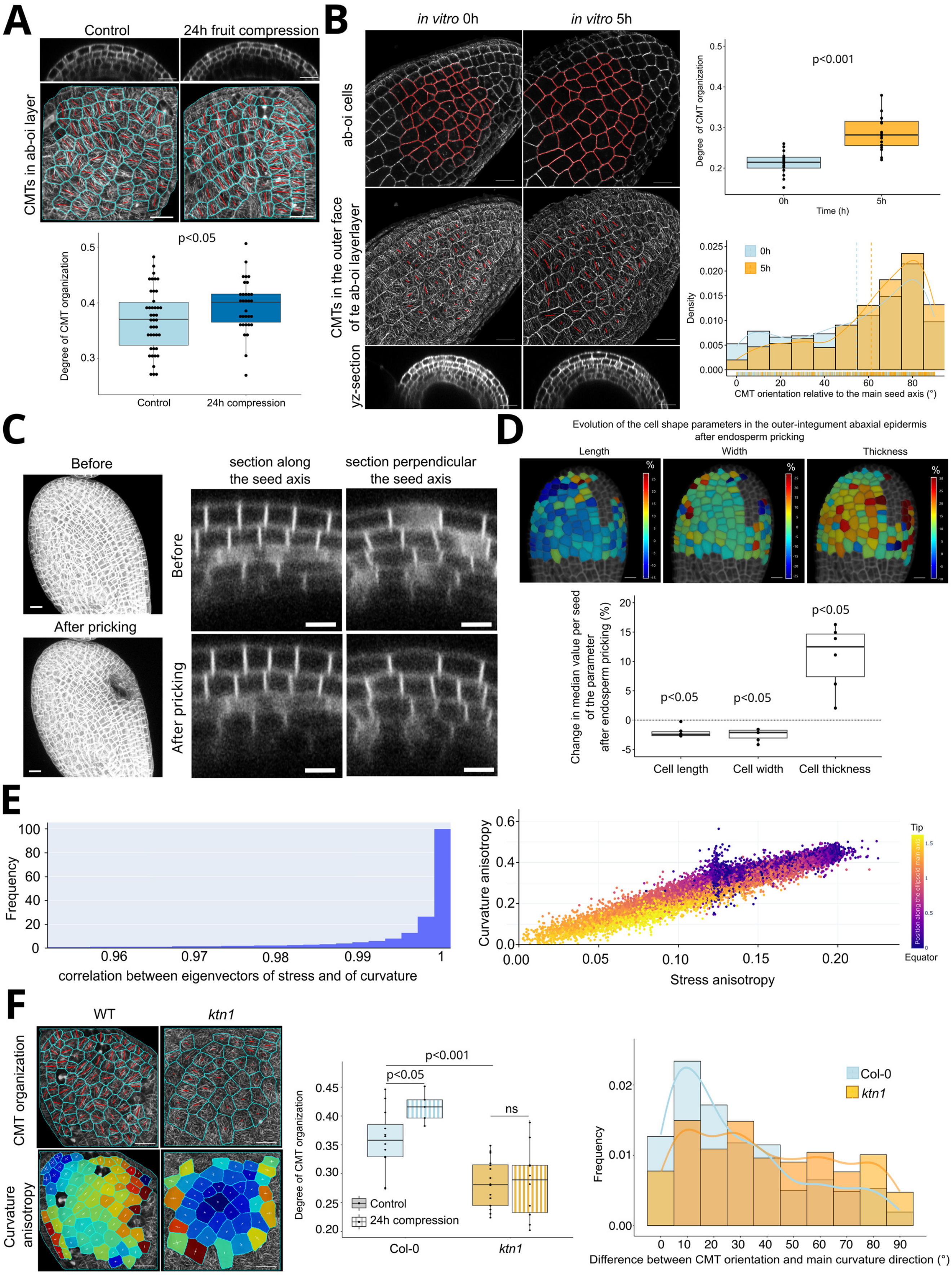
A
Control
24h fruit compression
CMTs in ab-oi layer
Degree of CMT organization
p<0.05
Control
24h compression
B
in vitro 0h
in vitro 5h
ab-oi cells
CMTs in the outer face of te ab-oi layerlayer
yz-section
p<0.001
Degree of CMT organization
Time (h)
0h
5h
Density
CMT orientation relative to the main seed axis (°)
C
Before
After pricking
section along the seed axis
section perpendicular the seed axis
D
Evolution of the cell shape parameters in the outer-integument abaxial epidermis after endosperm pricking
Length
Width
Thickness
Change in median value per seed of the parameter after endosperm pricking (%)
p<0.05
Cell length
Cell width
Cell thickness
E
Frequency
correlation between eigenvectors of stress and of curvature
Curvature anisotropy
Stress anisotropy
Tip
Equator
Position along the ellipsoid main axis
F
WT
ktn1
CMT organization
Curvature anisotropy
Degree of CMT organization
p<0.001
p<0.05
ns
Control
24h compression
Col-0
ktn1
Frequency
Difference between CMT orientation and main curvature direction (°)

Figure EV4. CMTs in the outer face of the outer integument abaxial epidermis align according to shape-driven stresses.

(**A**) Organization of the CMTs (imaged using the *p35S::MAP65-1-RFP* reporter) in the outer face of abaxial outer integument epidermis during the anisotropic growth phase (2DPA) in control seeds or in seeds whose fruits were compressed for 24 h with a microvice (following the protocol of Creff et al, 2015), $n = 1512–2319$ cells from 23 to 32 seeds, five independent experiments. In the pictures, the orientation of the red bars shows the mean orientation of the CMTs in each cell and its length shows its degree of organization. Data were compared using a bilateral Student test. In the boxplot representations, the midline represents the median of the data while the lower and upper limits of the box represent the first and third quartile respectively. The error bars represent the distance between the median and one and a half time the interquartile range. (**B**) Effect of a 5 h cultivation of developing seeds at 2DPA in liquid culture medium on the growth (representative pictures) and the organization of the CMTs facing the outer face (representative pictures and quantification) of the cells of the outer integument abaxial epidermis, $n = 741–751$ cells from 16 seeds, two independent experiments. Data were compared using a bilateral Student test. Scale bars, 10 µm. In the boxplot representations, the midline represents the median of the data while the lower and upper limits of the box represent the first and third quartile, respectively. The error bars represent the distance between the median and one and a half time the interquartile range. (**C**) Representative z-projections and sections parallel and perpendicular to the main seed axis of 2DPA seeds (imaged using the ubiquitous membrane marker *LTi6b-GFP*) showing the effect of a release of endosperm pressure by pricking on the aspect of seed coat cells. Scale bars, projections: $n = 20$ µm, sections: 10 µm. (**D**) Quantification of the median changes in length, width and height of the cells in the outer integument abaxial epidermis following endosperm pricking (see "Methods"), $n = 669$ cells from 6 seeds, two independent experiments. Scale bars, 10 µm. Data were compared to 0 using Wilcoxon tests. In the boxplot representations, the midline represents the median of the data while the lower and upper limits of the box represent the first and third quartile, respectively. The error bars represent the distance between the median and one and a half time the interquartile range. (**E**) Correlation between main stress direction and main curvature direction, and between stress anisotropy and curvature anisotropy based on the computational simulations shown in Fig. 4C and described in Appendix Supplementary Methods. (**F**) Quantification of the correlation between the main orientation of the CMTs and the main direction of curvature, and of the degree of organization of the CMTs following a 24 h compression, of WT and *ktn1* seeds at 2DPA. In the CMT pictures, the orientation of the red bars shows the mean orientation of the CMTs in each cell and their length, their degree of organization. In the heatmaps of curvature anisotropy, the orientations of the two perpendicular white bars represent the axes of maximum and minimum curvature in each cell and their lengths the degree of curvature in each of these two directions. Data were compared using bilateral Student tests, Curvature maps: Col-0: $n = 407–763$ cells from 6 to 14 seeds, *ktn1*: $n = 437–1008$ cells from 10 to 17 seeds, four independent experiments; compressions: Col-0 : $n = 723$ cells from 13 seeds, *ktn1* : $n = 934$ cells from 15 seeds, four independent experiments. Scale bars: 20 µm. In the boxplot representations, the midline represents the median of the data while the lower and upper limits of the box represent the first and third quartile, respectively. The error bars represent the distance between the median and one and a half time the interquartile range.

© The Author(s)

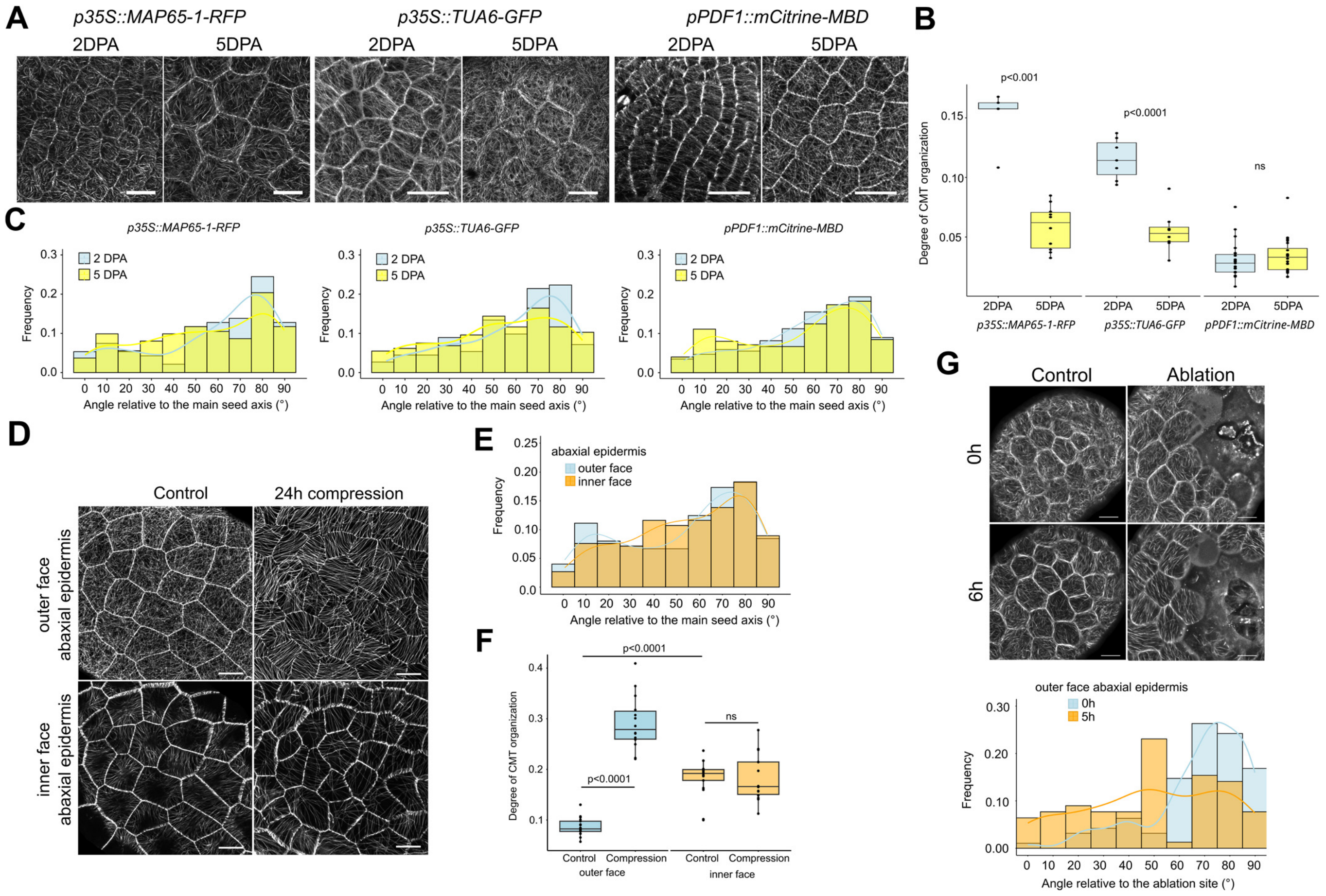

Figure EV5. CMT organization and response to forces in the outer integument abaxial epidermis during the isotropic growth phase.

(**A–C**) Comparison of the organization and orientation relative to the main seed axis of the CMTs in the outer face of the abaxial outer integument epidermis during the anisotropic growth phase (2DPA) and during the isotropic growth phase (5DPA) imaged using three different CMT reporters (*p35S::MAP65-1-RFP, p35S::TUA6-GFP, pPDF1::mCitrine-MBD*), scale bars: 20 µm, $n = 94$–491 cells from 5 to 23 seeds, two to three independent experiments depending on the reporter. Data were compared using bilateral Student tests. In the boxplot representations, the midline represents the median of the data while the lower and upper limits of the box represent the first and third quartile, respectively. The error bars represent the distance between the median and one and a half time the interquartile range. (**D**) Organization of the CMTs array facing the inner of the outer face of the seed in the abaxial epidermis at 5DPA (imaged using the *pPDF1::mCitrine-MBD* reporter) following a 24 h compression of the seed. Scale bars: 20 µm. (**E**) Quantification of the orientation relative to the main seed axis of the CMTs facing the inner and the outer face of the seed in the abaxial epidermis at 5DPA (imaged using the *pPDF1::mCitrine-MBD* reporter), $n = 273$ cells from 18 seeds. (**F**) Effect of a 24 h compression of the fruit on the degree of organization of the CMTs (imaged using the *pPDF1::mCitrine-MBD* reporter) facing the inner or the outer side of the seed in the abaxial outer integument epidermis at 5DPA, $n = 273$–293 cells from 14 to 18 seeds, two independent experiments. Data were compared using bilateral Student tests. In the boxplot representations, the midline represents the median of the data while the lower and upper limits of the box represent the first and third quartile, respectively. The error bars represent the distance between the median and one and a half time the interquartile range. (**G**) Effect of cell ablations on the orientation of the CMTs (imaged using the *p35S::MAP65-1-RFP* reporter) facing the outer face of 5DPA seeds in the abaxial outer integument epidermis, $n = 78$–95 cells of 12 to 13 seeds, scale bars: 20 µm, three independent experiments.

 © The Author(s)
